# Long-range inputome of cortical neurons containing corticotropin-releasing hormone

**DOI:** 10.1038/s41598-020-68115-x

**Published:** 2020-07-22

**Authors:** Peilin Zhao, Mengting Zhao, Huading Wang, Tao Jiang, Xueyan Jia, Jiaojiao Tian, Anan Li, Hui Gong, Xiangning Li

**Affiliations:** 1 Britton Chance Center for Biomedical Photonics, Wuhan National Laboratory for Optoelectronics, MoE Key Laboratory for Biomedical Photonics, School of Engineering Sciences, Huazhong University of Science and Technology, Wuhan, 430074 China; 2 HUST-Suzhou Institute for Brainsmatics, JITRI Institute for Brainsmatics, Suzhou, 215123 China; 3 CAS Center for Excellence in Brain Science and Intelligence Technology, Chinese Academy of Science, Shanghai, 200031 China

**Keywords:** Neural circuits, Cellular neuroscience

## Abstract

Dissection of the neural circuits of the cerebral cortex is essential for studying mechanisms underlying brain function. Herein, combining a retrograde rabies tracing system with fluorescent micro-optical sectional tomography, we investigated long-range input neurons of corticotropin-releasing hormone containing neurons in the six main cortical areas, including the prefrontal, somatosensory, motor, auditory, and visual cortices. The whole brain distribution of input neurons showed similar patterns to input neurons distributed mainly in the adjacent cortical areas, thalamus, and basal forebrain. Reconstruction of continuous three-dimensional datasets showed the anterior and middle thalamus projected mainly to the rostral cortex whereas the posterior and lateral projected to the caudal cortex. In the basal forebrain, immunohistochemical staining showed these cortical areas received afferent information from cholinergic neurons in the substantia innominata and lateral globus pallidus, whereas cholinergic neurons in the diagonal band nucleus projected strongly to the prefrontal and visual cortex. Additionally, dense neurons in the zona incerta and ventral hippocampus were found to project to the prefrontal cortex. These results showed general patterns of cortical input circuits and unique connection patterns of each individual area, allowing for valuable comparisons among the organisation of different cortical areas and new insight into cortical functions.

## Introduction

Corticotropin-releasing hormone (CRH), also known as corticotropin-releasing factor, is a 41-amino acid neuropeptide widely distributed in the central nervous system^1^. CRH containing neurons plays an indispensable role in maintaining individual health and homeostasis^2^ and is involved in stress^3^, learning, memory^4,5^ and social activity^6^. Abnormal CRH expression is associated with certain diseases, including Alzheimer’s disease^7^, depression, and post-traumatic stress disorder^8^. The cerebral cortex is an inalienable part of the central CRH system^9,10^, where CRH directly modulated postsynaptic depolarisation of pyramidal neurons^11,12^ and induces corresponding functional actions^13^. CRH plays various roles in the different cortical areas. For instance, those in the sensorimotor cortex contribute to depressant-like function by modulating motor activity during stress ^14^, whereas overexpression of CRH in the frontal cortex decreases locomotor and exploratory activity and produces an anxiolytic-like effect^15^. Interestingly, recent studies^16^ have verified the key role of CRH neurons in the medial prefrontal cortex (mPFC) in controlling behavioural-style selection during various stressful challenges.

Although CRH neurons in the cortex are GABAergic neurons that mainly project locally^17,18^, their activity is regulated via various circuits^19^ and they participate in different functions. Due to limited techniques for tracing and imaging, previous studies have mainly focused on local connections. By combining the modified rabies virus (RV) tracing system with Cre line mice^20^, recent studies have uncovered the long-range input circuits of CRH neurons in the anterior cingulate cortex (ACC) and have investigated their monosynaptic connections with the cholinergic system^21^. However, these results are based on two-dimensional sections which lack whole-brain information, especially anatomical information in three-dimensional (3D) space. Though it is unclear whether the input circuit of CRH neurons in other cortical areas have the same pattern.

Via the newly developed whole brain imaging systems^22,23^, we are able to investigate 3D information of the whole brain with high resolution, which can be used to study the distribution and neuronal morphology with cytoarchitecture information. Recent studies have uncovered input circuits of different GABAergic neurons in the mPFC^24^ and the difference between the afferent circuits of GABAergic and glutamatergic neurons in different motor cortices^25^. However, it is unclear whether input circuits of CRH neurons are similar or if there are some of the same areas in the input circuits that regulate different cortical areas.

Herein, we used a modified RV tracing system to explore the monosynaptic input of CRH neurons in six different cortical regions. We acquired whole-brain datasets of these input neurons with high resolution at 0.32 × 0.32 × 2 μm^3^ to analyse characteristics of input neurons to different cortical areas. These results lay anatomical foundations for future studies of functional organisation patterns of cortical CRH circuits.

## Results

### Transsynaptic labeling monosynaptic input to cortical CRH neurons

**Figure 1:**
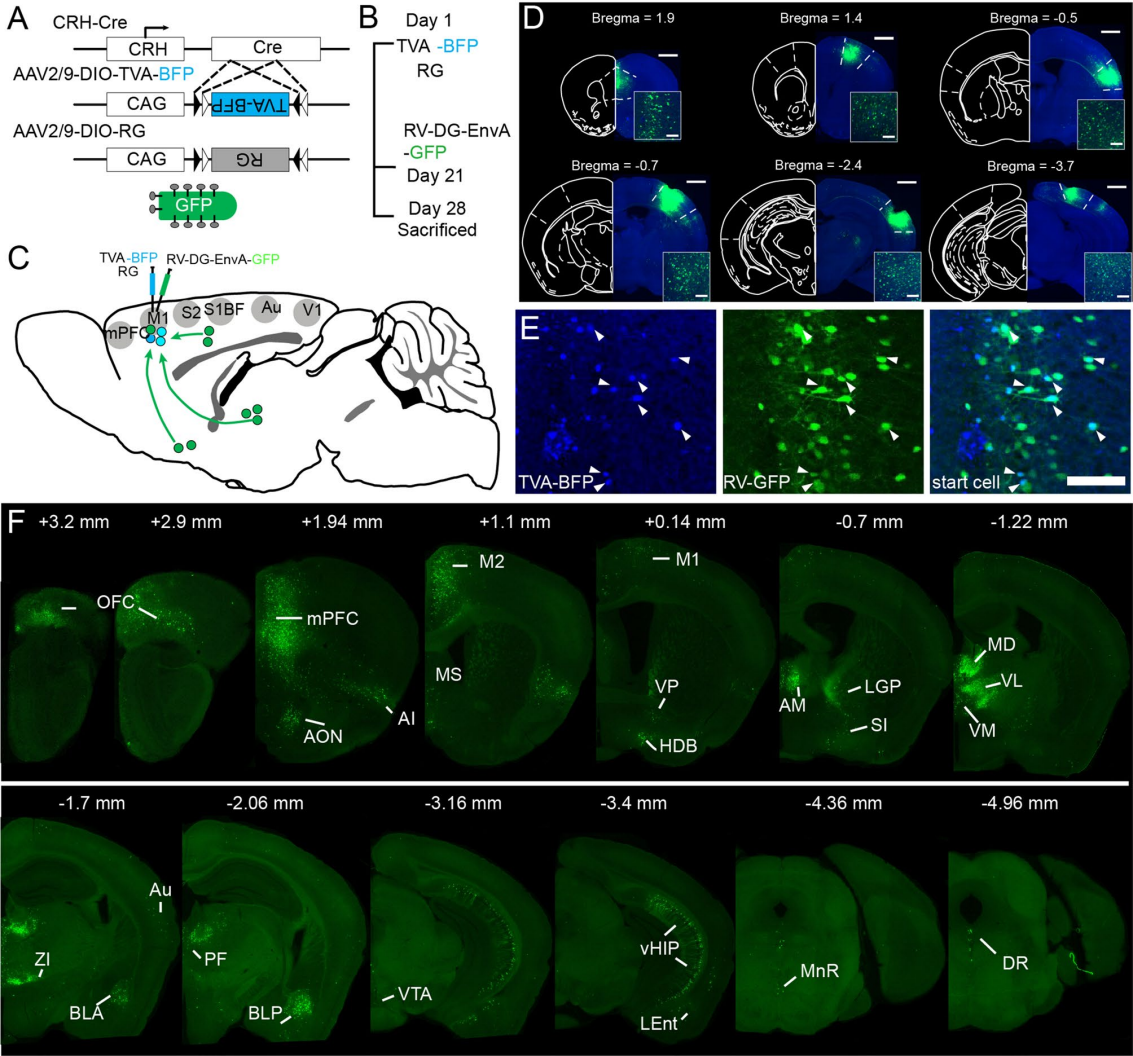
The acquisition of whole brain input to cortical CRH neurons. (**A**) CRH-Cre mice and helper virus express a fusion of TVA receptor and BFP and RG, then the modified RV virus uncover the whole brain input with GFP. (**B**) The time line of experimental operation. (**C**) Input of CRH mPFC, M1, S1BF, S2, Au and V1 were labeled. (**D**) Typical coronal plane of inject site, the start cells are confined in object regions, scale bar = 1000 μm, 200 μm respectively. (**E**) Clear view of start cells in mPFC. BFP labeled neurons are neurons infect by AAV helper, GFP labeled neurons are input neurons, the neurons co-labeled with BFP and GFP are start cells, scale bar = 200 μm. (**F**) Whole brain input of CRH mPFC. The abbreviations of brain regions are provided in Supplementary Table S2.

To map input neuron distribution patterns of cortical CRH neurons, modified RV tracing was performed on CRH-ires-Cre mice^10^. Firstly, we injected two Cre-dependent adeno-associated viruses (rAAV2/9-Ef1α-DIO-RG and rAAV2/9-Ef1α-DIO-His-BFP-TVA) into the cortex of CRH-Cre mice to co-express the TVA receptor and rabies virus G glycoprotein (RG) in cortical CRH neurons (Fig. 1A). Three weeks later, we injected RV (Fig. 1B) modified by an avian virus envelope protein (EnvA) in the same location. The gene expressing green fluorescent protein (GFP) replaced the endogenous G-glycoprotein gene of the RV. As the RV virus can only infect cells expressing TVA receptors, it was confined to CRH neurons expressing Cre recombinase. To compare the input pattern of different cortical regions, we labelled the CRH neurons in six cortical areas, including the mPFC, primary motor cortex (M1), barrel cortex (S1BF), secondary sensory cortex (S2), auditory cortex (Au) and primary visual cortex (V1) (Fig. 1C,D). As shown in Fig. 1E, the start cells were the neurons in injection sites labelled with GFP and BFP simultaneously, whereas neurons that only expressed GFP in different regions were input neurons (Fig. 1E,F, Supplementary Fig. S1). We detected the location of the start cells and found they were limited to injection sites but distributed throughout the different layers except layer 1 (Supplementary Fig. S2).

To examine the specificity of the virus tracing, we performed control experiments to verify the potential leakage expression of the virus. Firstly, we infected the mPFC of C57 mice with AAV-helpers and the M1 with saline. Three weeks later, we injected RV into these same areas. Seven days after the RV injection, the mice were perfused and 3 neurons (0, 1, and 2 neurons, respectively, n = 3 mice) were found in the mPFC (Supplementary Fig. S3A, B) whereas no neurons were labelled in the M1 (Supplementary Fig. S3C, D), indicating that the AAV-helper carrying TVA had a slightly leaky expression, which was consistent with previous studies^26^. Furthermore, previous studies^9,16^ have shown that CRH-Cre mice crossed with Cre-dependent reporter mice allowed for accurate characterisation of the distribution of CRH neurons. To detect leakage of the AAV-helper carrying BFP, we injected AAV-helpers in into the M1 of CRH-Cre:LSL-H2B-GFP mice, the soma of CRH neurons which express GFP. Two weeks later, the mice were perfused and 94 ± 2% of the BFP labelled neurons were found to co-express GFP (Supplementary Fig. S3E,F,G). These results show that the virus we used has a good specificity.

As previously reported^26^, leakage of the construct carrying TVA would result in putative overestimation of local presynaptic cells. Therefore, in the present work, we only analysed long-range input neurons.

### The whole brain distribution of input neurons to cortical CRH neurons

**Figure 2:**
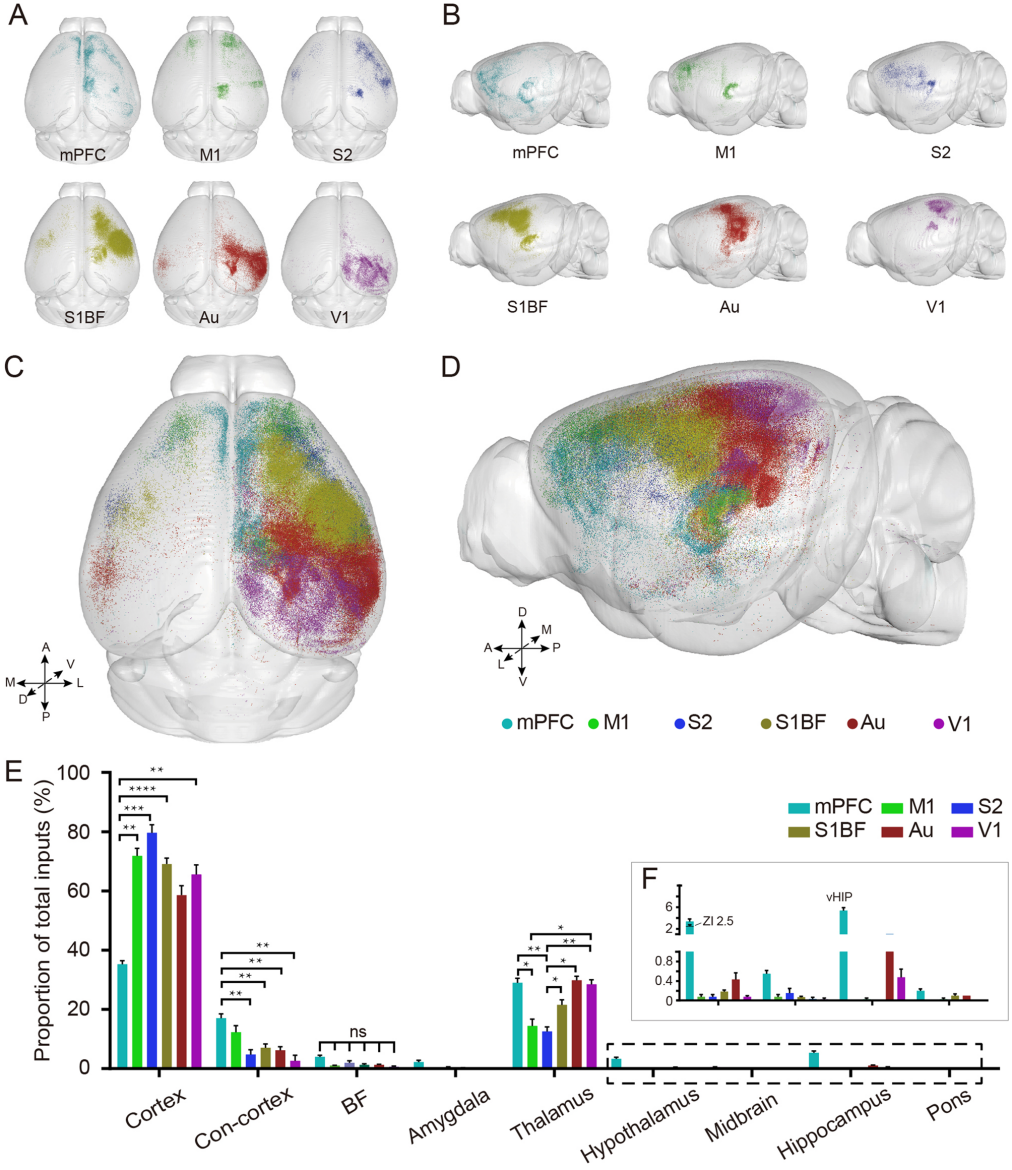
Quantification of whole brain input to cortical CRH neurons. (**A**) The planform of all six whole brain input datasets mapped to the Allen Reference Atlas in 3D. (**B**) The sagittal plane of all six whole brain input datasets mapped to the Allen Reference Atlas in 3D. (**C**) The planform of whole brain input to CRH neurons in different cortical areas. (**D**) The sagittal plane of whole brain input to CRH neurons in different cortical areas. (**E**) Quantitative statistical proportion of whole brain input. Histogram of different colors show the input of CRH neurons in different cortical areas. Con-cortex means Contralateral cortex. (**F**) A clear view of input in hypothalamus (ZI), midbrain, hippocampus (vHIP) and pons. (**A**–**D**) 3D vision displayed via Amira software (v5.2.2, Mercury Computer Systems, San Diego, CA, United States). Data shown as mean ± SEM. One-way ANOVA followed by Tukey’s post hoc tests, *P < 0.05, **P < 0.01, ****P < 0.0001; mPFC n = 4; M1 n = 4; S2 n = 4; S1BF n = 6; Au n = 4; V1 n = 4, for detailed P values, see Supplementary Table S3.

To uncover the organisation of the input circuits of cortical CRH neurons in the whole brain, we acquired the continuous dataset of input neurons at 0.32 × 0.32 × 2 μm^3^ with fluorescence micro-optical sectioning tomography (fMOST) serial technologies^22,23^. To quantify the distribution of input neurons, the datasets were reconstructed and registered to the Allen Brain Reference Atlas. Input patterns among the different samples were then compared. As shown in Fig. 2A,B, we obtained and reconstructed the spatial distribution of input neurons to CRH neurons in six cortical areas, including the mPFC, M1, S1, S1BF, Au and V1 respectively. We found that the input neurons were widely distributed, ranging from the orbitofrontal cortex (OFC) to the midbrain and pons. These input regions have various predilections to the six cortical areas, among which anterior regions projected to anterior cortical areas (mPFC, M1 and S2) and posterior regions projected to posterior cortical areas (S1BF, Au and V1) (Supplementary Fig. S1). The input neurons were mainly located in the ipsilateral hemisphere of the injection site, with a few found in the contralateral cortex.

To accurately compare input differences of the different cortical areas in the whole brain, we put the input neurons of different cortical regions into the same formulate as the reference atlas^27^. Different colour dots represent input neurons of the six different cortical areas (Fig. 2C,D). The horizontal and coronal view showed the distribution pattern of the input neurons. Although the input neurons of these cortical areas were mainly distributed in the ipsilateral hemisphere, they had different distribution patterns in that they preferred to locate around injection sites. Input neurons in the mPFC and M1 were more dispersed compared to those in the V1 (Supplementary Fig. S1).

To quantify the difference among these input neurons, we counted with reference to the Paxinos’ atlas^28^ and the Allen Reference Atlas (ARA)^27^ . Input neurons were located in the cortex, thalamus, basal forebrain (BF), amygdala, hypothalamus, hippocampus, midbrain, and pons (Fig. 2E, Supplementary Table S1). According to quantitative statistics, long-range afferent neurons were mainly found in the ipsilateral cortex (mPFC, 38.0 ± 2.4%; M1, 73.0 ± 5.6%; S2, 79.0 ± 6.2%; S1BF, 69.9 ± 6.7%; Au, 53.9 ± 11.7%; V1, 65.2 ± 6.6%), followed by the thalamus (mPFC, 28.6 ± 3.9%; M1, 15.1 ± 5.4%; S2, 12.6 ± 3.7%; S1BF, 22.8 ± 5.9%; Au, 36.6 ± 8.6%; V1, 29.3 ± 3.4%), the contralateral hemispheric cortex (mPFC, 16.0 ± 3.1%; M1, 11.8 ± 5.0%; S2, 5.2 ± 3.7%; S1BF, 6.9 ± 3.8%; Au, 5.8 ± 1.9%; V1, 4.4 ± 3.9%), and the BF (mPFC, 4.8 ± 2.0%; M1, 1.1 ± 0.3%; S2, 2.1 ± 1.5%; S1BF, 1.1 ± 0.9%; Au, 1.9 ± 0.7%; V1, 0.7 ± 0.3%), with only a few input neurons located in the mesencephalon and pons (Fig. 2E). In addition, a great number of neurons were found in the zona incerta (ZI, 2.6 ± 0.8%) and ventral hippocampus (vHIP,5.2 ± 1.4%) and formed monosynaptic connections with CRH neurons in the mPFC (CRH_mPFC_) (Fig. 2F).

To better understand input patterns of CRH neurons in different cortical areas, the number of input neurons in a discrete brain region was normalised to the total number of input neurons. Percentages of the main input regions were compared with One-way ANOVA followed by Tukey’s post hoc tests. Results showed that CRH_mPFC_ received less input from the ipsilateral cortex and more from the contralateral cortex than those in M1, S2, S1BF, and V1. CRH neurons in these six cortical areas received similar inputs from BF, wheras S2 received the least input from the thalamus (Fig. 2E and Supplementary Table S3). These results suggest that CRH neurons in different cortical areas receive divergent input from the cortex and thalamus in distinct patterns.

### Quantitation of cortico-cortical inputs

**Figure 3:**
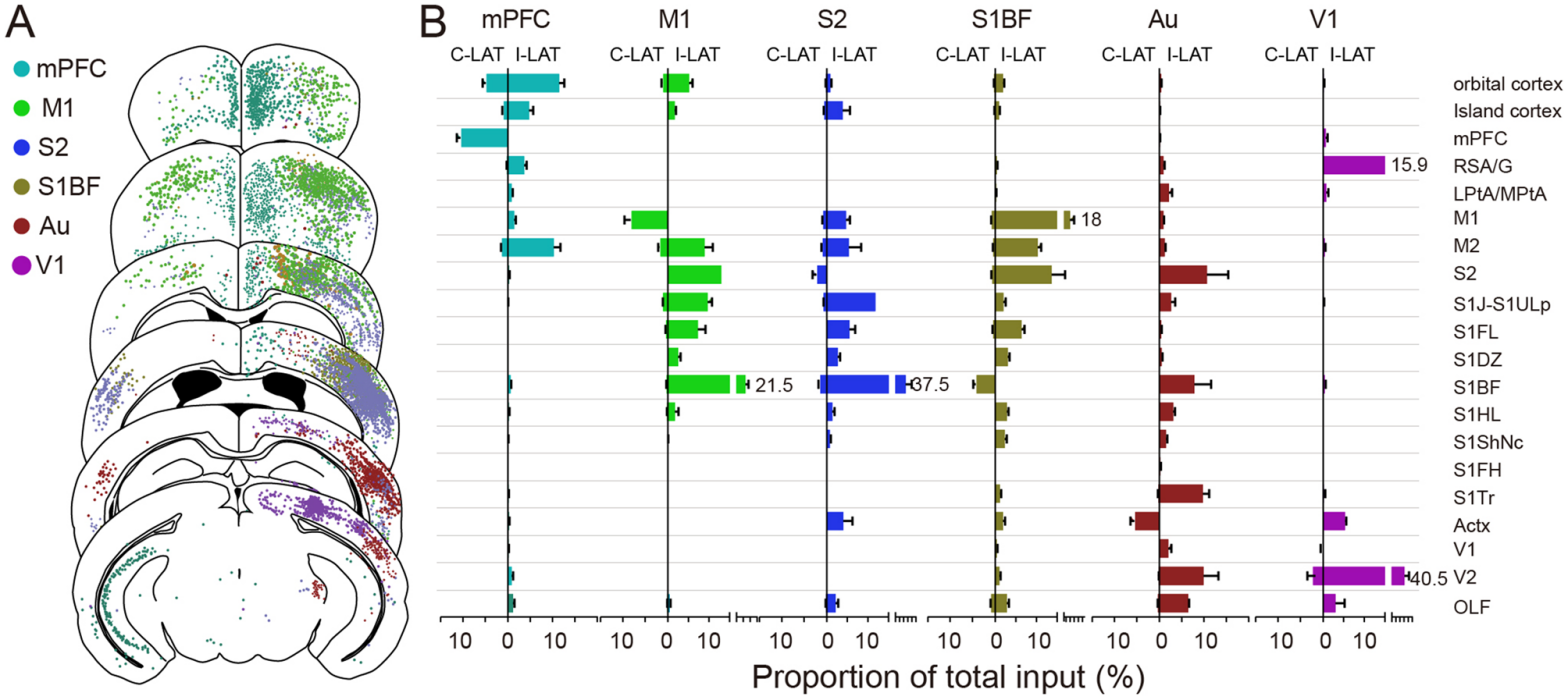
Cortico-cortical input to CRH neurons. (**A**) Continuous coronal view for the distribution of afferent neurons in cortex. Each dot present one neuron while different color shows input to CRH neurons in different cortical areas. (**B**) The ratio of cortical input neurons to whole brain input. The left side of the vertical axis represents the proportion of input neurons in the contralateral cortex of the injection site, and the right side represents ipsilateral cortex. Data shown as mean ± SEM. mPFC n = 4; M1 n = 4; S2 n = 4; S1BF n = 6; Au n = 4; V1 n = 4. The abbreviations of brain regions are provided in Supplementary Table S2.

The neocortex is the advance centre of brain evolution^29^. Cortical CRH neurons received direct input from varying cortical areas (Fig. 3A). To comprehensively analyse the distribution of cortical input neurons, we counted afferent neurons in 20 different cortical regions (Fig. 3B) and found that cortical CRH neurons mainly received input from adjacent cortical regions. For CRH_mPFC_, afferent neurons were mainly from the OFC and M2. For M1, input neurons gathered in the sensory cortex (S2, S1J, S1FL, S1BF) and M2. For S2, most cortical input neurons were found in the motor and primary sensory cortex (S1J, S1BF). For S1BF, afferent neurons converged in the motor cortex and S2, and for Au, input neurons were mainly located in the sensory cortex (S2, S1BF, S1Tr), V2, and perirhinal area. Afferent neurons of V1 were mainly from the V2 and retrosplenial cortex (RSA/G). Interestingly, CRH neurons in these six cortical areas all received monosynaptic input from the ipsilateral OFC and perirhinal area, and the mPFC, M1, and sensory cortex simultaneously received direct input from insular cortex neurons, while neurons in the RSA/G stimulated mPFC and V1 CRH neurons (Fig. 3).

Regarding the contralateral cortex, input neurons were primarily located in the contralateral homonymous cortex. Additionally, the OFC formed direct monosynaptic connections with the contralateral mPFC, M1, S1BF, S2, and Au, none of which were afferent to the contralateral V1. The perirhinal area directly projected to the contralateral M1, S1BF, S2, and Au, however, not to the mPFC or V1. The mPFC received simultaneous input from the contralateral insular cortex and M2, whereas the M1 received a small amount of input from the contralateral M2 and S1. The sensory cortex received additional input from the contralateral motor cortex (Fig. 3B).

These results show that neurons in the OFC and perirhinal area form wide connections with CRH neurons in diverse cortical areas in both hemispheres and in the anterior cortex (mPFC and M1) received more input from the contralateral cortex than posterior cortical areas (Au and V1).

### Region specificity of thalamic input

**Figure 4:**
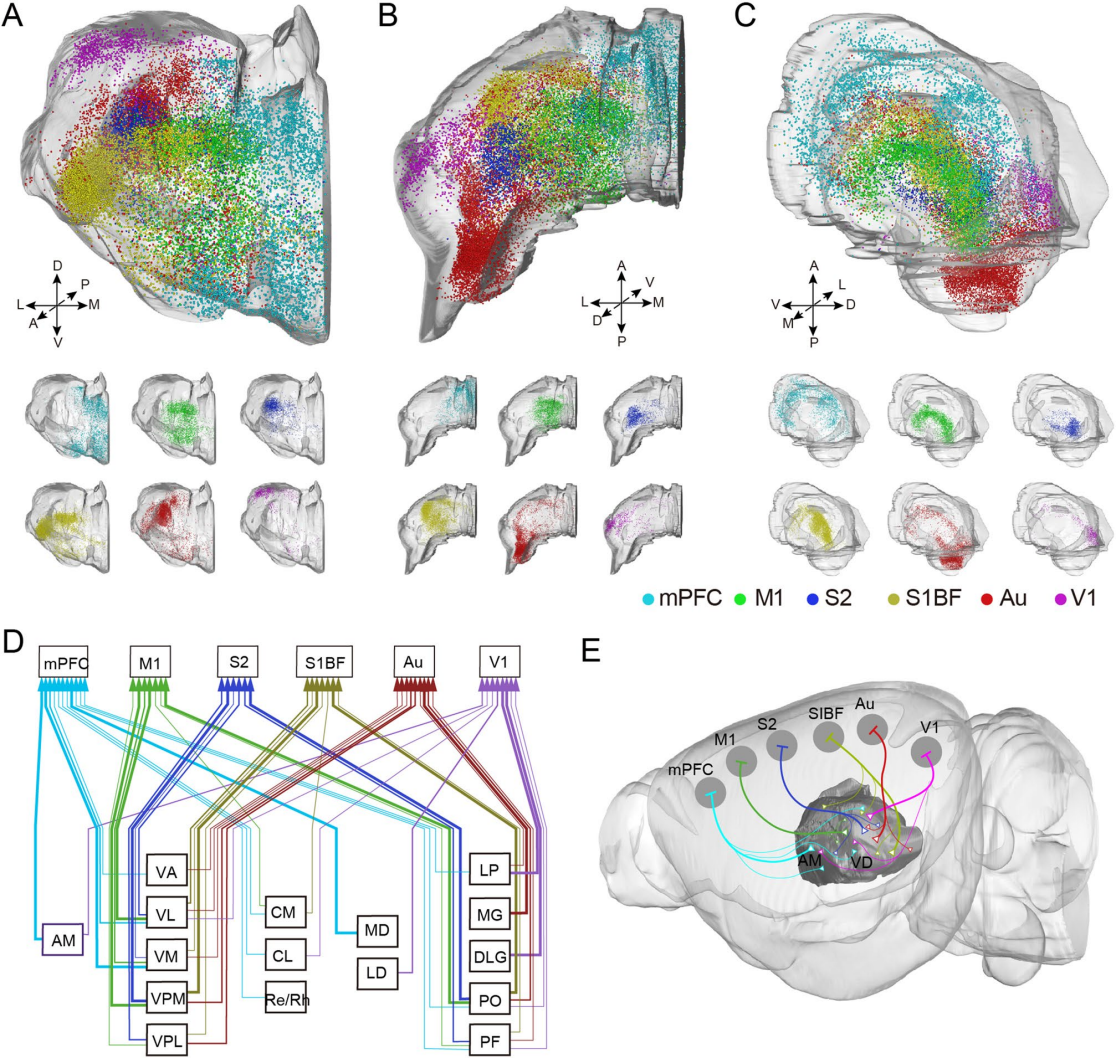
Region-specific distribution of thalamic inputs to cortical CRH neurons. (**A**) The front 3D view of input neurons in thalamus. (**B**) The planform 3D view of input neurons in thalamus. (**C**) The lateral 3D view of input neurons in thalamus. The top one is the over view of afferent neurons input to CRH neurons in different cortical areas. The six below are input neurons to mPFC, M1, S2, S1BF, Au and V1, respectively. (**D**) The monosynaptic relationship and intensity between the thalamic nuclei and cortical CRH neurons. Polylines in different color represent the input to different cortical CRH cortex, and the thick arrows indicate dense distribution of input neurons. (**E**) The diagram of connection between cortical CRH neurons and thalamus. (**A**,**B**,**C**,**E**) 3D vision displayed via Amira software (v5.2.2, Mercury Computer Systems, San Diego, CA, United States).The abbreviations of brain regions are provided in Supplementary Table S2.

The thalamus is an important sensory conduction substitution station in the central nervous system. Various whole-body sensory conduction pathways exchange information in the thalamus then target the cerebral cortex. As one of the main afferent resources of cortical CRH neurons, thalamic projection neurons occupied more than 20% of whole-brain long-range input neurons (Fig. 2E). Our previous work^25^ found that thalamic neurons projected to different motor cortices located in different subregions of the thalamus. For further certification of the difference among input neuron distribution patterns of these six cortical areas, we 3D reconstructed the thalamus and located the input neurons into the outlines together. From Fig. 4A–C, we found that the anterior thalamic nuclei mainly projected to the CRH neurons in the rostral cortex, while the posterior thalamic nuclei preferred the caudal cortex (Supplementary Fig. S4). Furthermore, thalamic nuclei near the midline favoured the rostral cortex, whereas the lateral thalamic nuclei preferred the caudal cortex.

To further investigate input neurons in the thalamus, we compared their density in different thalamic nuclei and found that monosynaptic afferents to CRH_mPFC_ gathered in the anteromedial thalamic nucleus (AM), mediodorsal thalamic nucleus (MD), and the anterior ventrolateral thalamic nucleus (VL), whereas the M1 mainly received inputs from the posterior VL and the anterior part of the posterior thalamic nuclear group (PO). The input to S1BF and S2 gathered in different parts of the ventral posteromedial thalamic nucleus (VPM) and posterior PO. For Au, input mainly come from the medial geniculate nucleus (MG), while for V1, input was mainly located in the dorsal lateral geniculate (DLG) and lateral posterior thalamic (LP) nuclei (Fig. 4D,E). In addition, we found the VL in the ventral thalamus, PO, and parafascicular thalamic nucleus (PF) and the PO had monosynaptic connections with various cortical CRH neurons. Interestingly, the central medial thalamic nucleus (CM), centrolateral thalamic nucleus (CL), reuniens thalamic nucleus, and rhomboid thalamic nucleus (Re/Rh) which are near the midline projected to cortical CRH neurons relatively close to the midline, but not to the lateral cortex S2 and Au. additionally, we found that the thalamic nucleus in the peripheral part (AM in anterior; MD and LD in dorsal; Re/Rh in ventral; MG and DLG in posterior) connected with CRH neurons in specific cortical areas, while the inner thalamic nucleus had divergent connections with different cortices.

### Wide distribution of inputs from basal forebrain

**Figure 5:**
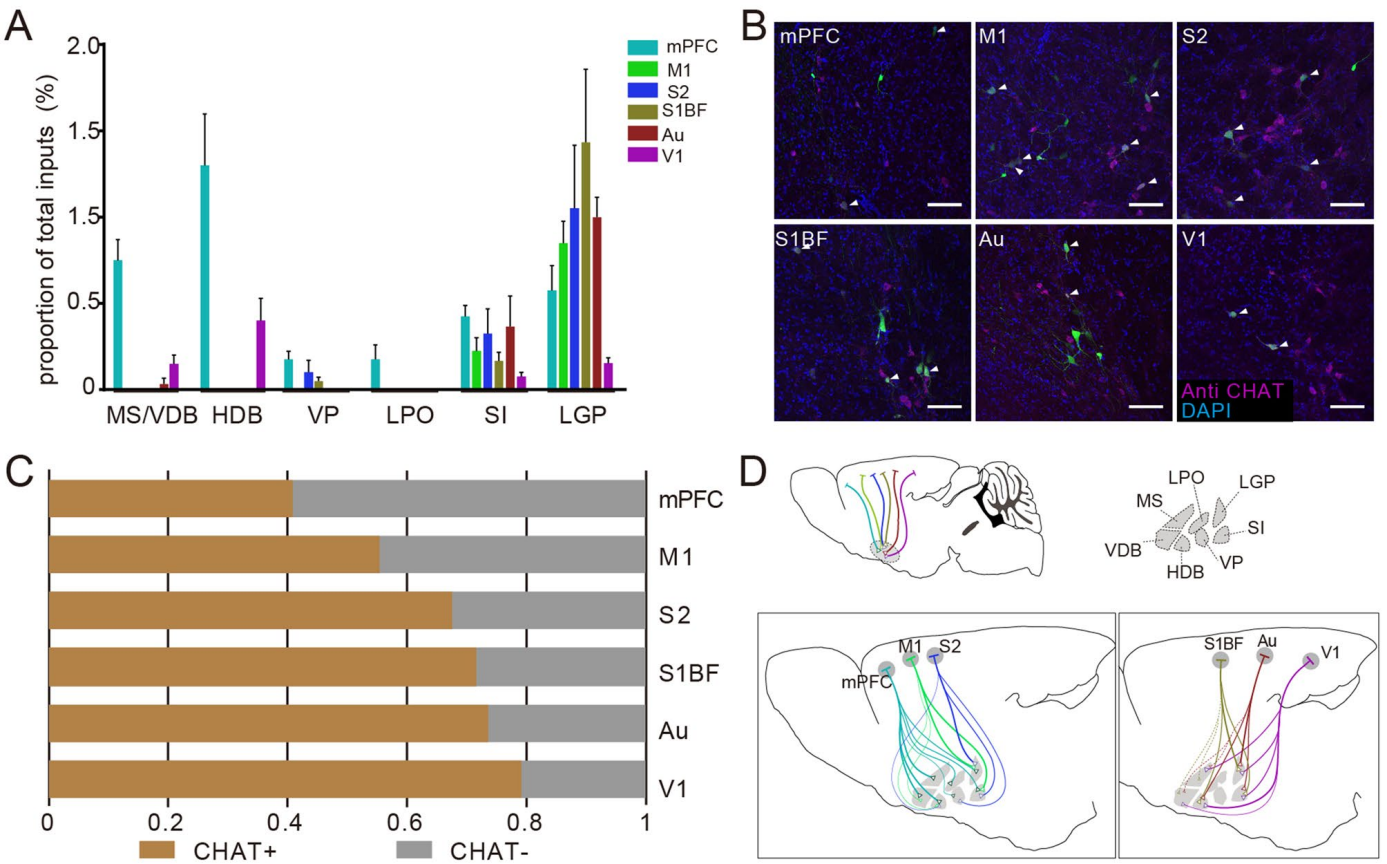
Inputs in the basal forebrain to cortical CRH neurons. (**A**) The proportion of input neurons in different subregions of the BF. Different color represent input to CRH neurons in different cortical areas; Data shown as mean ± SEM; mPFC n = 4 mice; M1 n = 4 mice; S2 n = 4 mice; S1BF n = 6 mice; Au n = 4 mice; V1 n = 4 mice. (**B**) The immunofluorescent staining of cholinergic neurons in LGP. The arrows point to input neurons merged with cholinergic neurons, scale bar = 100 μm. (**C**) Proportion of cholinergic neurons input to cortical CRH neurons in BF. (**D**) A diagrammatic drawing of BF cholinergic neurons projected to cortical CRH neurons. The abbreviations of brain regions are provided in Supplementary Table S2.

The BF is another major subcortical afferent region of cortical CRH neurons (Fig. 1D). From Fig. 5A, we found that neurons in different subregions of the BF stably contacted different cortical areas. Input neurons clustered in the substantia innominata (SI) and lateral globus pallidus (LGP) except for V1, which was preferred by nuclei of the horizontal limb of the diagonal band (HDB). Furthermore, CRH_mPFC_ received rich input from nuclei of the vertical limb of the diagonal band (VDB) and HDB. Previous studies have shown that the BF is a region enriched with cholinergic neurons with a wide range of fibre projections^30^. To further investigate the relationship between the cholinergic system and cortical CRH neurons, we identified input neurons in the BF via immunofluorescent staining. Consistent with the distribution of input neurons in the BF, cholinergic positive input neurons were mainly located in the SI and LGP (Fig. 4B, Supplementary Fig. S5). As shown in Fig. 5C, we found that over half of the input neurons in the BF were cholinergic positive except for the mPFC, and that the ratio of cholinergic positive input neurons was higher for cortical CRH neurons in the caudal cortex. Interestingly, CRH_mPFC_ received strong cholinergic input from VDB and HDB, whereas the V1 the majority of cholinergic positive input neurons gathered in the HDB rather than in the SI or LGP (Fig. 4D).

## Discussion

Determining how long-range inputs engage cortical neurons is crucial in understanding circuit mechanisms in regulating cortical functions. Using the RV tracer system and the whole-brain imaging system, we mapped a whole-brain atlas of input neurons to cortical CRH neurons. The distribution of input neurons to the six cortical areas had similar patterns in that most were in the ipsilateral cortex, while some in contralateral cortex, thalamic nucleus, and BF. In these afferent regions, input neurons of different subregions preferred CRH neurons in specific cortical areas (Figs. 3B, 4A–E, 5A,D).

The cortex can be divided into several regions according to characteristics and functions. To describe the whole brain input distribution of cortical CRH neurons as a whole, we selected six regions including the mPFC, M1, S1BF, S2, Au, and V1 (Fig. 1D), which covered the main cortical areas. We acquired whole-brain input datasets via fMOST, allowing us to distinguish long-range input neurons at a single-cell level. We obtained spatial location information of every input neuron according to continuous datasets with high resolution and reconstructed whole-brain inputs to CRH neurons in different cortical areas in the Allen brain CCF to study distribution pattern characteristics of input neurons. As per previous reports, there were strong connections between the different cortical areas^31^, critical for information processing. In line with the cortical input to other GABAergic neurons^24,25,32^, the cortical afferent neurons of the CRH_mPFC_ were mainly located in the OFC, M2, and contralateral mPFC, whereas for the M1 they were located in the sensory and contralateral motor cortex, and for S1BF in the motor cortex and S2 (Fig. 3).

As for subcortical input, the CRH_mPFC_ received similar monosynaptic afferent input to other GABAergic neurons which were mainly from the vHIP, thalamus, and BF (Fig. 2E,F). Cortical CRH neurons were certified as GABAergic neurons and co-expressed with VIP and SST partly^10,18^. However, it’s worth noting that abundant neurons in the ZI (Fig. 2F) were found targeting CRH_mPFC_ that was not explicitly describe in other studies of GABAergic neurons in this region, including PV, SST, and VIP positive neurons^32^. Thus, we could hypothesis that CRH_mPFC_ is involved in feeding^33,34^ and other functions closely related to the ZI.

Thalamocortical circuits are indispensable for sensory information processing in the cortex and play an important role in multiple functions, including attention^35^, motor learnin^36^, cognitive control^37^, perception of sounds^38^, and arousal^39^. Thalamocortical circuits showed topological connection that different subregions preferred different cortical neurons^25,40^. In this study, we labelled the thalamic input circuits of cortical CRH neurons and rebuilt them in line with the spatial location information we had acquired (Fig. 4A–C). We found a connection pattern in that the anterior and middle thalamus preferred the rostral cortex, while the posterior and lateral thalamus projected to the caudal cortex. Meanwhile, the thalamus nuclei near the midline projected to the cortical neurons relatively close to the midline but not to the lateral cortex (Fig. 4A–C). These region specific connections may help us predict the thalamic input to CRH neurons in other cortical areas. Furthermore, we found that the thalamic nuclei in the lateral part had convergent connections with CRH neurons in specific cortical areas, while the inner thalamic nuclei had divergent connections with different cortices, indicating that the inner thalamus may collect more complex information and modulate the activity of neurons in diverse cortical areas.

Neurons in in BF were in close contact with the cortex. As the indispensable source of acetylcholine arriving at cortex, cholinergic neurons in the BF (CNBF) modulate the activity of cortical neurons by abundant fibres^41^ and are involved in various functions, including cognitive operations^42,43^, goal-directed behaviour and reward^44–46^, motor skill learning^47^, cortical plasticity^48–50^, auditory processing and sound related learning^51,52^, and visual perception^53–55^. The CNBF projected to CRH neurons in the ACC^21^ directly and could change the release of cortical CRH^19^. In this study, we uncovered the organisation of cholinergic neurons afferent to cortical CRH neurons via immunofluorescence staining and found cholinergic positive input neurons widely distributed in different subregions of the BF, most of which were located in the LGP and SI (Fig. 5B). To our surprise, CRH neurons in the V1 were preferred by the cholinergic neurons in HDB (Fig. 5D). It’s worth noting that over half of the input neurons in the BF were cholinergic positive except for CRH_mPFC_ (Fig. 5C), indicating that the BF may regulate cortical CRH neurons mainly through cholinergic neurons. As stress is a complex physiological response involving a variety of behaviours, and both CRH neurons and CNBF contribute to stress^16,56^, we suppose that the regulation of CNBF on CRH neurons in different cortical regions may participate in various functions related to stress.

In conclusion, we mapped the whole brain input of cortical CRH neurons with modified retrograde RV. We examined the distribution of the monosynaptic input neurons of CRH neurons in different cortical areas and the thalamus and verified the single synaptic connection to BF cholinergic neurons. This study lays a structural foundation for the study of functional loops related to cortical CRH neurons.

## Materials and Methods

### Animals

All animal experiments in this study were approved by the Institutional Animal Ethics Committee of Huazhong University of Science and Technology and all experiments were performed in accordance with relevant guidelines and regulations. Adult male CRH-Cre mice (2–4 months), C57BL/6J and CRH-Cre:LSL-H2B-GFP mice were used in the study. CRH-Cre transgenic mice (Stock No: 012704) and GFP reporter LSL-H2B-GFP mice (Stock No: 032577) were purchased from The Jaxon Laboratories. CRH-Cre:LSL-H2B-GFP mice were generated by crossing CRH-Cre male mice with LSL-H2B-GFP female mice. CRH-Cre mice were used to label the input neurons. Three CRH-Cre:LSL-H2B-GFP mice and three C57BL/6J mice were used as controls. The mice were housed in a 12-h light/dark cycle condition with free access to sufficient food and water.

### Viruses information

In this study, we used two adeno-associated viruses (AAV) as helpers and one modified RV. The titer of rAAV2/9-Ef1α-DIO-RG and rAAV2/9-Ef1α-DIO-His-BFP-TVA both were 2–5 × 10^12^ genome copies/mL and the RV-EnvA-DG-GFP was 2.5 × 10^8^ international units/mL. All viral tools were packed by BrainVTA (BrainVTA Co., Ltd., Wuhan, China).

### Surgery and viral injections

Mice were anaesthetised with mixed anaesthetics (2% chloral hydrate and 10% ethylurethane dissolved in 0.9% NaCl saline) according to their weight (0.1 mL/10 g) before viral injection. The heads of the anesthetised mice were fixed with a stereotaxic holder to adjust skull position. A cranial drill (~ 0.5 mm diameter) was used to penetrate the skulls above target areas. 100 ~ 150 nL of AAV helper (mixed with rAAV2/9-Ef1α-DIO-oRVG and rAAV2/9-Ef1α-DIO-His-BFP-TVA in a ratio of 1:2) was injected into the mPFC (AP 1.94 mm, ML 0.3 mm, DV − 2.3 mm), M1 (AP 1.4 mm, ML 1.65 mm, DV − 1.6 mm), S2 (AP − 0.5 mm, ML 3.75 mm, DV − 2.85 mm), S1BF (AP − 0.7 mm, ML 2.85 mm, DV − 1.75 mm), Au ( AP  − 2.46 mm, ML 4.05 mm, DV − 2.25 mm), and V1 (AP − 3.7 mm, ML 2.3 mm, DV − 1.0 mm) in different mice in about 10 min. Three weeks later, 200–250 nL of RV-EnvA-DG-GFP was injected into the same location via the same methods mentioned above.

### Histology and immunostaining

The histological operations followed previous studies^57^. One week after RV injection, anesthetised mice were perfused with 0.01 M PBS (Sigma-Aldrich, United States), followed with 2.5% sucrose and 4% paraformaldehyde (PFA, Sigma-Aldrich, United States) in 0.01 M PBS. The brains were removed and post-fixed in 4% PFA solution overnight.

To detect labelled signals and characterize input neurons in the BF, some samples were sectioned in 50 μm coronal slices with a vibrating slicer (Leica 1200S). Immunofluorescent staining was done on 19 ± 5 sections (mPFC, 22, 23 slices; M1, 14, 14 slices; S2, 18, 19 slices; S1BF, 18, 22 slices; Au, 14, 15 slices; V1,18, 21 slices) selected from each brain for each inject site containing labelled neurons in the BF. These slices were blocked with 0.01 M PBS containing 5% bovine serum albumin (BSA, Sigma–Aldrich, United States)) and 0.3% Triton X-100 for 1 h at 37 °C. The sections were incubated with the primary goat anti-chat monoclonal antibody (1:500, Abcam AB144P) overnight at 4 °C and washed in PBS 5 times at room temperature. They were stained with Alexa-Fluor 594, donkey anti-goat (1:500, Abcam, ab150132) for 1.5 h at 37 °C. After rinsing with PBS, DAPI (1 ng/mL) was performed on tinct sections for 5 min, washed, and then mounted. We acquired the stained information of the sections with a confocal microscope (LSM 710, Zeiss, Jena, Germany).

### Imaging and statistical analysis

For whole-brain imaging, brain samples were dehydrated with alcohol and embedded with GMA resin, and information acquired with the brain-wide positioning system (BPS)^23^. Briefly, the embedded sample was fixed on the base and the image of the top surface acquired in two simultaneous fluorescent channels, the imaged tissue was subsequently removed via slicing. Thus, we could obtain continuous whole brain dataset layer by layer at a voxel resolution of 0.32 × 0.32 × 2 μm^3^.

For cell counting, we collected 50 μm sections from 26 brains and every second slice was imaged with confocal microscopy (Zeiss LSM710). The images were manually registered to the Paxinos’ atlas^28^ in 2D according to cytoarchitectonic information, and imported the images to the Fiji software and manually counted the labelled cells with its cell counter module^25^. All input areas except for injection sites were counted. For adjacent areas with dense neurons, we divided the neurons on the junction into the left or upper brain regions.

For statistical analysis, the number of input neurons in a discrete brain region was normalised to the total number of input neurons. Statistical graphs were generated using GraphPad Prism v.8.02 and Microsoft Excel (Office 2019). One-way ANOVA followed by Tukey’s post hoc tests were performed via GraphPad Prism v. 8.02. The confidence level was set to 0.05 (P value) and all results were presented as the means ± SEM. The data distribution was tested with a Shapiro–Wilk test (α = 0.05).

### 3D visualization

To compared input distribution of CRH neurons in different cortical areas in 3D, we registered the whole-brain datasets to the Allen Reference Atlas^58^ and compared input neurons in the thalamus. The methods of registration have been previously described^59^. Briefly, image preprocessing was performed to correct uneven illumination and remove background noise. The down sampling data (the voxel resolution of 10 µm × 10 µm × 10 µm) was uploaded into Amira software (v5.2.2, Mercury Computer Systems, San Diego, CA, United States) to distinguish and extract regional features of anatomical invariants, including the outline of brain, the ventricles, the corpus callosum, and the corpus striatum, etc. Next, the current advanced grey-level based registration algorithm (SyN) was used to register the extracted features and obtain corresponding relationships between the image dataset and the Allen CCFv3 brain atlas. To analyse and display the input organisation in 3D, spatial information of the cell body was extracted from the whole-brain data by NeuroGPS software^60^ and placed in the corresponding 3D brain region outline. Basic operations including extraction of areas of interest, resampling and maximum projection were performed via Amira software and Fiji (NIH).

## Supplementary information

Supplementary file1 (PDF 1144 kb)

## Data Availability

The data that support the findings of this study are available on https://atlas.brainsmatics.org for readers to access and download.
