# Supplementary material for: Long-range inputome of cortical neurons containing corticotropin-releasing hormone
Source: Sci Rep. 2020 Jul 22;10:12209. doi: 10.1038/s41598-020-68115-x (PMC7376058; doi:10.1038/s41598-020-68115-x)
Supplement: Supplementary file 1 — Supplementary file1 (PDF 1144 kb) [file 41598_2020_68115_MOESM1_ESM.pdf]

Long-range inputome of cortical neurons containing corticotropin-releasing hormone

Peilin Zhao[1], Mengting Zhao[1], Huading Wang[1], Tao Jiang[2], Xueyan Jia[2], Jiaojiao Tian[1], Qingtao Sun[1], Anan Li[1, 2, 3], Hui Gong[1, 2, 3]*, Xiangning Li[1, 2]*

[1] Britton Chance Center for Biomedical Photonics, Wuhan National Laboratory for Optoelectronics, MoE Key Laboratory for Biomedical Photonics, School of Engineering Sciences, Huazhong University of Science and Technology, Wuhan 430074, China

[2] HUST-Suzhou Institute for Brainsmatics, JITRI Institute for Brainsmatics, Suzhou 215123, China

[3]CAS Center for Excellence in Brain Science and Intelligence Technology, Chinese Academy of Science, Shanghai 200031, China

Correspondence

Xiangning Li, lixiangning@mail.hust.edu.cn

Hui Gong, huigong@mail.hust.edu.cn

Supplementary Figure S1 Overview of whole-brain input distribution

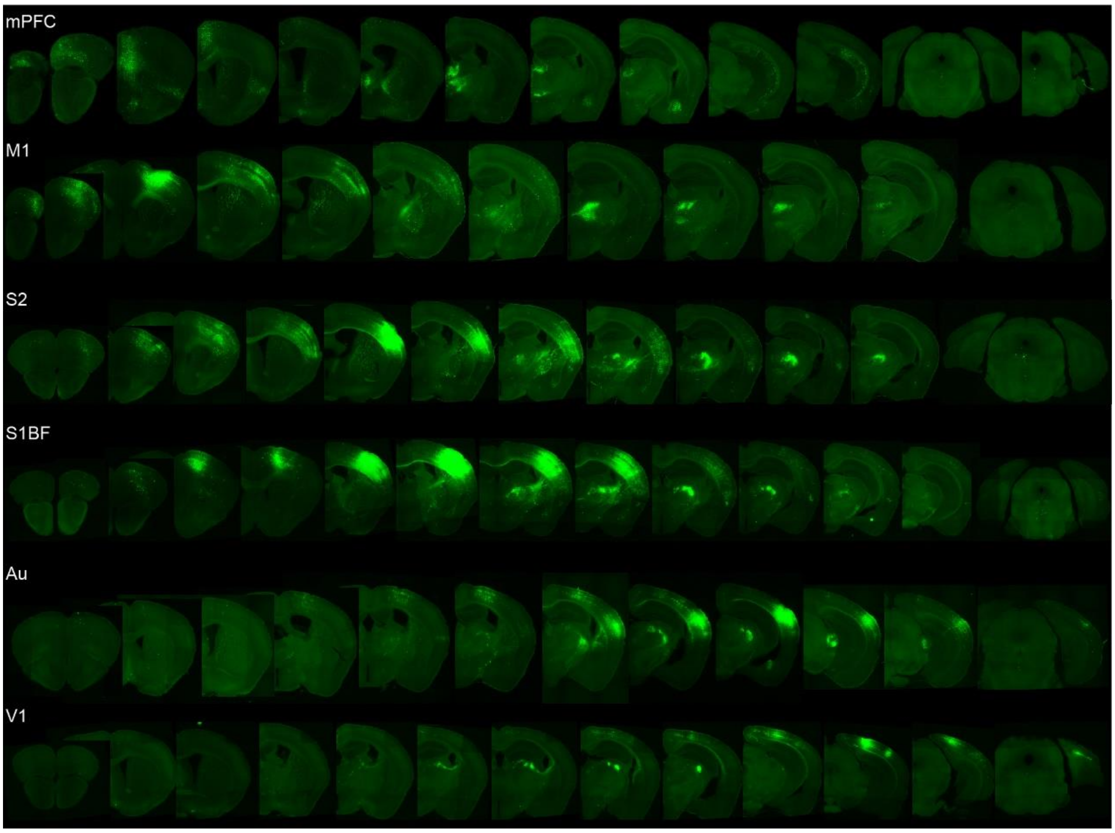

From top to bottom represents whole-brain input to mPFC, M1, S2, S1BF, Au and V1, respectively.

Supplementary Figure S2 The location of start cells

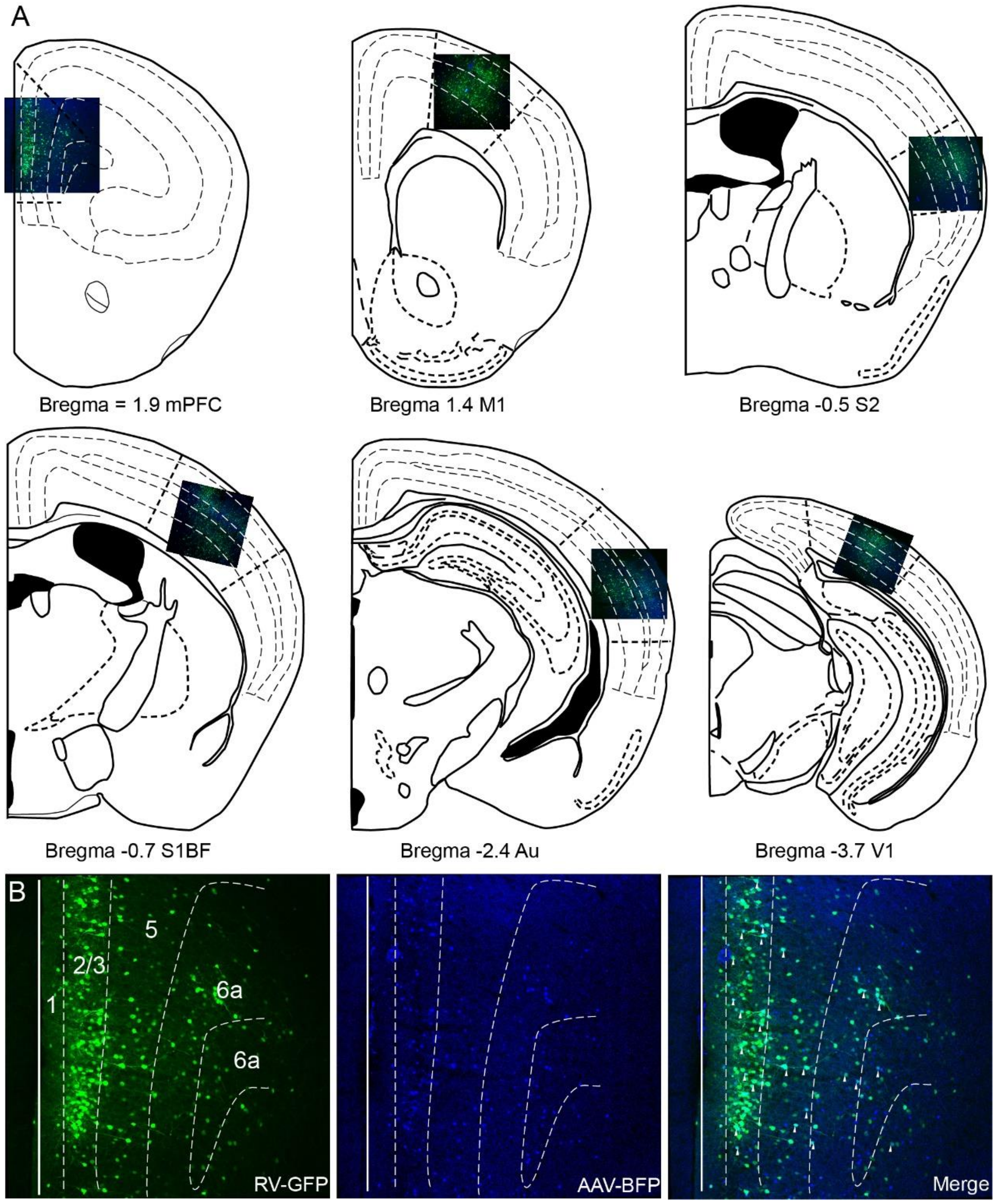

(A) The location of inject site in atlas. Start cells were confined to inject area. (B) A three-panel presentation with RV, BFP and merge, arrows point out start cells co-labeled with GFP and BFP. Start cells distributed in different layers of mPFC except layer 1.

Supplementary Figure S3 The specificity of virus used

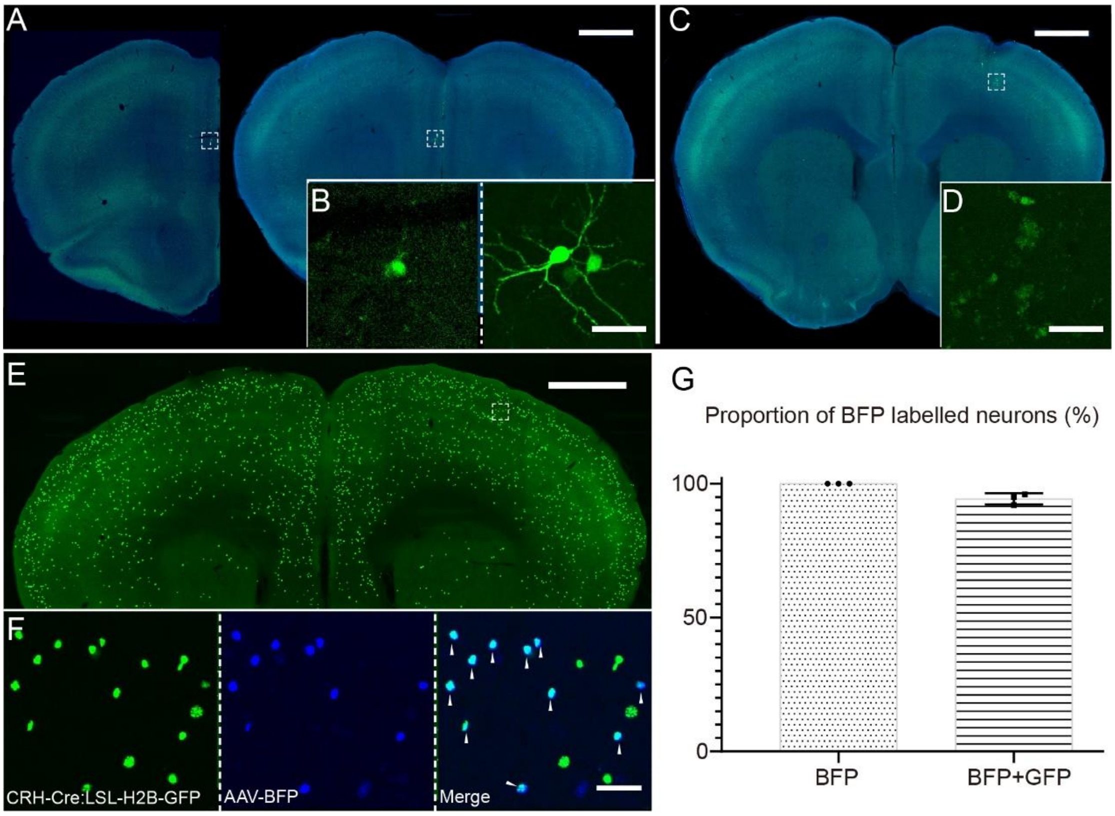

(A) AAV helpers were injected in mPFC of C57 mice and RV was performed in the same site three weeks later. (B) Clear view of the labelled neurons. (C) RV was performed in the M1 directly. Scale bar=1000μm. (D) An enlarged view of inject site, not any labeled neurons were found in M1. (E) AAV-DIO-TVA-BFP was performed in M1of CRH-Cre:LSL-H2B-GFP mice. (F) A three-panel presentation with CRH neurons, BFP and merged. Arrows point out co-labelled neurons. (G) The proportion of neurons co-labelled with BFP and GFP. Data shown as mean ± SEM; n = 3 mice. (A) (C) (E)Scale bar = 1000μm. (B) (D) (F) Scale bar = 50μm.

Supplementary Figure S4 The distribution of thalamic input neurons

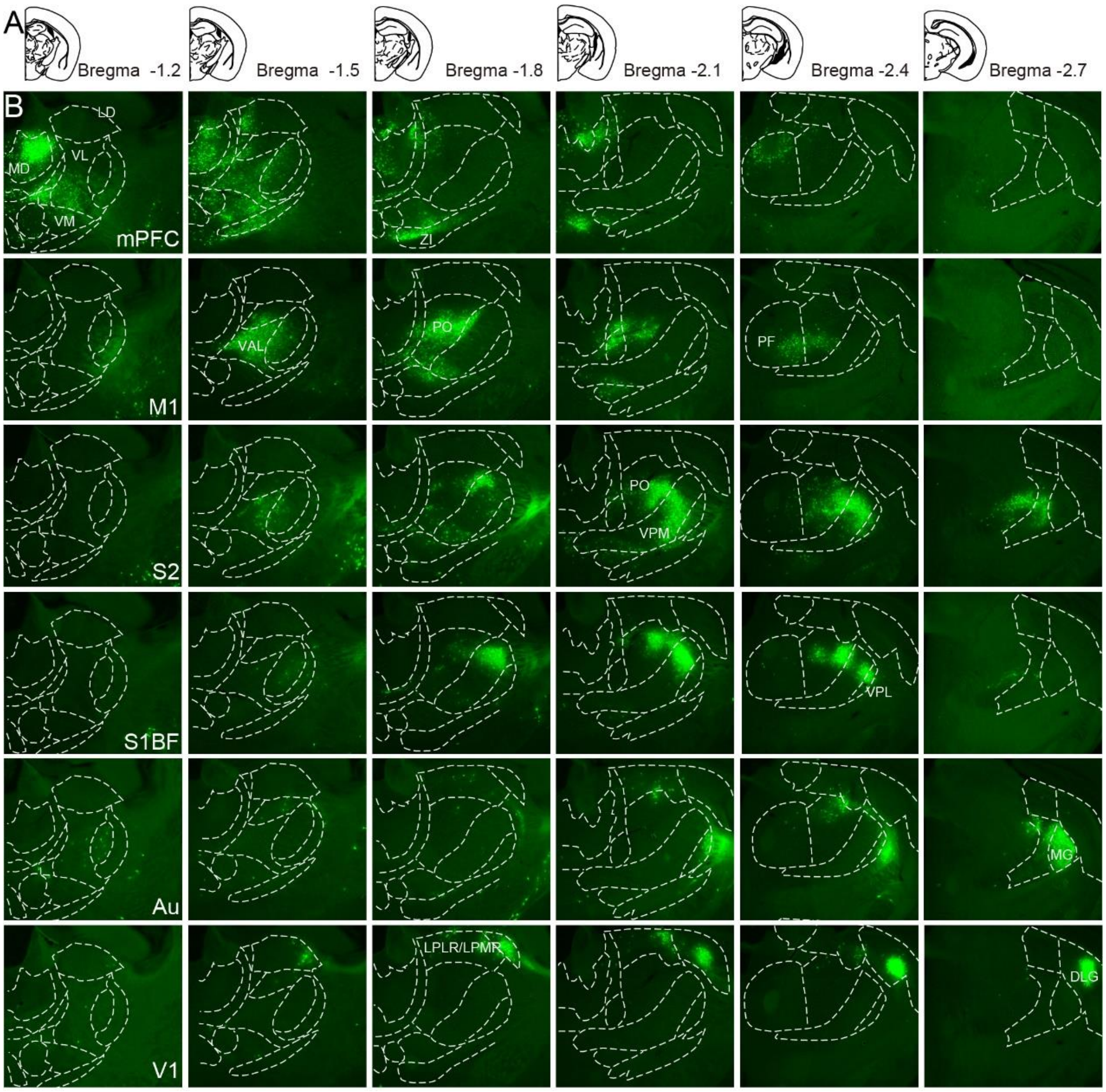

(A) The coronal plane in which different subregions of the thalamus are located. (B) The distribution of thalamic afferent neurons. From top to bottom represents thalamic input to mPFC, M1, S2, S1BF, Au and V1, respectively.

Supplementary Figure S5 The cholinergic positive input neurons in the BF

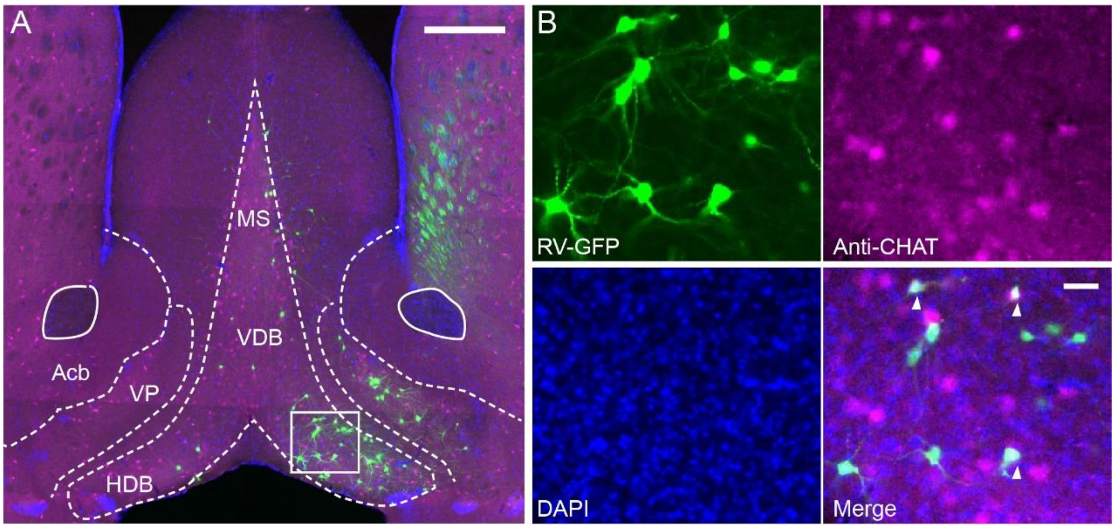

(A) Immunohistochemical staining of the BF neurons input to mPFC, Scale bar=500μm. (B) A four-panel presentation with RV, anti-CHAT, DAPI and merged, arrows point out cholinergic positive input neurons, scale bar = 50μm.

Supplementary Table S1 The number of cells counted

	cortex	con-cortex	BF	amygdaloid	thalamus	hypothalamus	hippocampus	Middle brain	pons	total
**mPFC**	3418	1232	642	128	3110	374	570	62	10	9546
	4371	2338	565	243	3397	601	668	87	27	12297
	5776	2688	572	596	3703	568	982	70	33	14988
	5376	2488	370	225	3923	314	506	67	27	13296
**M1**	7696	671	131	2	1264	11	0	3	3	9781
	5604	885	90	9	1701	15	0	9	0	8313
	7119	1543	117	6	1523	5	0	17	2	10332
	7720	1788	83	20	1034	0	0	4	1	10650
**S2**	5638	98	38	4	840	2	0	0	0	6620
	7067	304	226	92	964	0	0	32	0	8685
	7508	508	328	8	824	8	0	0	0	9184
	6634	807	117	39	1486	7	0	10	9	9109
**S1BF**	7944	581	23	30	1757	10	0	13	3	10361
	7595	355	240	10	3325	25	0	5	15	11570
	4349	737	131	32	1618	1	0	4	12	6884
	5072	334	148	12	1532	8	0	2	2	7110
	5982	710	111	36	1784	18	0	13	20	8674
	5833	856	89	9	1498	13	0	10	5	8313
**Au**	2916	346	101	2	1417	13	49	1	5	4850
	4356	261	81	11	1860	12	60	2	5	6648
	3285	448	69	3	1881	11	74	4	6	5781
	2328	393	147	6	2493	15	114	6	6	5508
**V1**	2554	47	40	0	933	4	28	0	0	3606
	2829	399	18	0	1581	6	0	0	0	4833
	2894	24	20	0	1063	1	26	1	1	4030
	2360	16	37	0	1025	2	17	3	0	3460

Supplementary Table S2 The abbreviation of brain regions

Abbrevition	Full name
OFC	orbitalfrontal cortex
mPFC	medial prefrontal cortex
AON	anterior olfactory nucleus
AI	agranular insular cortex
M1	primary motor cortex
M2	secondary motor cortex
S2	secondary somatosensory cortex
S1Tr	primary somatosensory cortex, trunk region
S1FH	primary somatosensory cortex, forelimb region
S1ShNc	primary somatosensory cortex, shoulder/neck region
S1HL	primary somatosensory cortex, hindlimb region
S1BF	primary somatosensory cortex, barrel field
S1DZ	primary somatosensory cortex, dysgranular region
S1FL	primary somatosensory cortex, forelimb region
S1J-S1ULp	primary somatosensory cortex, jaw region/upper lip region
Au	auditory cortex
LPtA/MPtA	lateral/medial parietal association cortex
RSA/G	retrosplenial agranular / granular cortex
LEnt	lateral entorhinal cortex
V1	primary visual cortex
V2	secondary visual cortex
vHIP	ventral hippocampus
BLA	basolateral amygdaloid nucleus, anterior part
BLP	basolateral amygdaloid nucleus, posterior part
MS/VDB	medial septal nucleus / nucleus of the vertical limb of the diagonal band
HDB/MCPO	nucleus of the horizontal limb of the diagonal band/magnocellular preoptic nucleus
LPO	lateral preoptic area
VP	ventral pallidum
SI	substantia innominata
LGP	lateral globus pallidus
AM	anteromedial thalamic nucleus
VA	ventral anterior thalamic nucleus

VL	ventrolateral thalamic nucleus
VM	ventromedial thalamic nucleus
VPM	ventral posteromedial thalamic nucleus
VPL	ventral posterolateral thalamic nucleus
CM	central medial thalamic nucleus
CL	centrolateral thalamic nucleus
Re/Rh	reuniens thalamic nucleus / rhomboid thalamic nucleus
MD	mediodorsal thalamic nucleus
LD	laterodorsal thalamic nucleus
LP	lateral posterior thalamic nucleus
MG	medial geniculate nucleus
DLG	dorsal lateral geniculate nucleus
PO	posterior thalamic nuclear group
PF	parafascicular thalamic nucleus
ZI	zona incerta
VTA	ventral tegmental area
MnR	median raphe nucleus
DR	dorsal raphe nucleus

Supplementary Table S3 Detail P values of significance analysis

		mPFC	M1	S2	S1BF	Au	V1
input from cortex	mPFC	——	P=0.0011	P=0.0006	p< 0.0001	ns	P=0.0054
	M1	P=0.0011	——	ns	ns	ns	ns
	S2	P=0.0006	ns	——	ns	ns	ns
	S1BF	p< 0.0001	ns	ns	——	ns	ns
	Au	ns	ns	ns	ns	——	ns
	V1	P=0.0054	ns	ns	ns	ns	——
input from con-cortex	mPFC	——	ns	P=0.0092	P=0.0097	P=0.0097	P=0.0082
	M1	ns	——	ns	ns	ns	ns
	S2	P=0.0092	ns	——	ns	ns	ns
	S1BF	P=0.0097	ns	ns	——	ns	ns
	Au	P=0.0097	ns	ns	ns	——	ns
	V1	P=0.0082	ns	ns	ns	ns	——
input from thalamus	mPFC	——	P=0.0352	P=0.0057	ns	ns	ns
	M1	P=0.0352	——	ns	ns	ns	P=0.0204
	S2	P=0.0057	ns	——	P=0.0323	P=0.0429	P=0.0024
	S1BF	ns	ns	P=0.0323	——	ns	ns
	Au	ns	ns	P=0.0429	ns	——	ns
	V1	ns	P=0.0204	P=0.0024	ns	ns	——
